# Bee venom loaded chitosan nanoparticles enhances growth, immunity and resistance to *vibrio parahaemolyticus* in pacific white shrimp

**DOI:** 10.1038/s41598-025-11011-z

**Published:** 2025-07-18

**Authors:** Moaheda E. H. Eissa, Basma M. Hendam, Noha I. ElBanna, Salah M. Aly

**Affiliations:** 1 Biotechnology Department, Fish Farming and Technology Institute, Suez Canal University, Ismailia, 41522 Egypt; 2 Department of Animal Wealth Development, Faculty of Veterinary Medicine, Mansoura University, Mansoura, 35516 Egypt; 3 Aquaculture Diseases Control Department, Fish Farming & Technology Institute, Suez Canal University, Ismailia, 41522 Egypt; 4 Rangeland, Wildlife and Fisheries Management Department, Texas A&M University, College Station, 77843 TEXAS United States; 5 Pathology Department, Faculty of Veterinary Medicine, Suez Canal University, Ismaillia, 41522 Egypt

**Keywords:** Apitoxin, Nano-chitosan, *Litopenaeus vannamei*, Immunity, Histopathology, Biotechnology, Genetics, Immunology, Zoology

## Abstract

Despite the known anti-inflammatory, antibacterial, and antioxidant properties of bee venom (apitoxin), its application for promoting growth and health in shrimp (Litopenaeus vannamei), remains largely unexplored. This study explored the effects of bee venom (BV) encapsulated nano-chitosan (BV-CSNPs) on the health and disease resistance of white shrimp. Two hundred forty shrimp (mean weight 6.0 ± 0.02 g) were randomly assigned to four groups (n = 60 per group), each consisting of four replicate hapa (1 m³ each) stocked with 20 shrimp. Shrimp were fed either a basal diet (control) or diets added with 0.1, 0.2, or 0.3 mg of bee venom encapsulated nano-chitosan (BV-CSNPs) per kg of feed for 63 days. Following the feeding trial, shrimp (n = 30 form each group) were challenged with Vibrio parahaemolyticus, and survival rates were subsequently assessed. Supplementation with BV-CSNPs in shrimp diets resulted in significantly enhanced weight gain and feed efficiency compared to the basal diet (P < 0.05). BV-CSNPs supplementation significantly increased hemocyte counts and phenoloxidase levels in a dose-dependent manner (P < 0.05). The highest dose (0.3 mg/kg) also significantly enhanced lysozyme and phagocytic activities compared to the other treatments (P < 0.05). Significant increases in the activities of antioxidant enzymes (SOD and CAT) and digestive enzymes (lipase, amylase and protease) were observed in all BV-CSNP-supplemented groups compared to the control group (P < 0.05). Regarding gene expression, a dose-dependent increase in the expression of immune genes associated with the proPO system (LGBP, PX, and ppA) and antioxidant genes (cytMnSOD and mtMnSOD) was observed in shrimp fed diets supplemented with BV-CSNP (0.1, 0.2, and 0.3 mg/kg). Histopathological analysis revealed normal hepatopancreas and intestinal tissue structure, with increased hepatopancreatic B-cell secretion and improved intestinal histomorphology in BV-CSNP-treated shrimp. These groups also exhibited lower mortality rates after the challenge with V. parahaemolyticus. Dietary inclusion of BV-CSNP proved highly effective in promoting shrimp growth and overall health. The benefits observed include enhanced digestive enzyme activity, improved intestinal integrity, robust antioxidant and innate immune responses, and effective protection against V. parahaemolyticus infection.

## Introduction

Aquaculture plays a vital role in sustainable food production, providing valuable products for global human consumption^1^. The increasing effects of global warming on water temperatures, marine biodiversity, and aquatic flora underscore the critical need to enhance fish farming practices^2^. Among farmed species, *Litopenaeus vannamei*, commonly known as the Pacific white shrimp, stands as the most important cultured species within the Penaeidae family. Its high commercial value, exceptional breeding ability^3^and adaptability to a wide range of salinities, coupled with the availability of specific pathogen-free (SPF) broodstock^4^, make it the dominant species in global shrimp aquaculture. Consequently, shrimp farming has emerged as a primary food production method in coastal regions, driving economic growth^5^and fostering small-scale entrepreneurial activity^6^.

The sustainability of *L. vannamei* farming is significantly threatened by various factors, dominant them the outbreaks of bacterial diseases. Intensification, suboptimal water quality, and undernutrition across different production stages exacerbate this issue, leading to global industry impacts^7,8^.

*Vibrio parahaemolyticus*, a Gram-negative, halophilic bacterium prevalent in marine, estuarine, and coastal environments, is a primary concern, causing substantial mortalities and significant economic losses worldwide^9,10^. Intensive aquaculture’s reliance on antibiotics for bacterial infection control presents a significant challenge. While crucial for disease management, widespread antibiotic use promotes drug-resistant bacteria, compromising future treatment efficacy. Moreover, most of administered antibiotics (70–80%) contaminate the environment, adversely affecting ecosystems and aquatic life^11,12^. Consequently, there is a growing imperative for sustainable, environmentally friendly bioactive materials that can enhance immunity and productivity for disease control, thereby overcoming the limitations of antibiotics^13^.

Nature provides a range of disease-mitigation strategies for aquaculture, such as microalgae, herbal extracts and oils, probiotics, and fungi. However, honeybee products have recently garnered significant attention as a promising alternative. Building on the suggestion that bee-derived compounds possess the potential to treat bacterial diseases in fish^14^ researchers have begun to investigate the utility of royal jelly, propolis, and pollen in aquaculture settings^15^. Bee venom (BV), or apitoxin, is a complex biotoxin produced by honeybees (family Apidae) in a specialized gland connected to their sting^16^. While primarily a defense mechanism against predators^17^, BV exhibits a wide array of pharmacological actions, including anti-inflammatory, antimutagenic, antibacterial, radioprotective, anti-nociceptive, immunostimulatory, hepatoprotective, and anti-cancer effects^18–20^. Notably, BV has demonstrated antimicrobial activity against several fish pathogens^21,22^, and its dietary supplementation significantly enhanced growth performance in Thinlip mullet, *Chelon ramada*^23^. Beyond individual benefits, BV also contributes to social antisepsis and collective immunity within the bee colony.

Chitosan, a naturally occurring compound, is widely recognized as a promising, environment friendly feed additive, finding applications across aquaculture, agriculture, medicine, food, and the chemical industry^24^. Its utility extends to drug delivery systems in various fields^25^. In aquaculture, chitosan supplementation in fish diets has significantly enhanced growth performance, immune function, and antioxidant status^26^. However, its nano-conjugation with BV remains a relatively unexplored area. The use of polymers like chitosan as drug carriers offers a novel approach in aquaculture, improving the stability, efficacy, and water solubility of natural compounds^27^.

While BV has applications in complementary medicine for treating inflammation-related diseases in humans^28^. and its antibacterial effects against bacteria from diverse sources have been studied^29,30^, its use in shrimp aquaculture, whether in natural or nanoparticle form, remains unexplored. To our knowledge, this study is the first to evaluate the dietary incorporation of bee venom as a nano-nutrient in shrimp feed, investigating its potential as both a nutritional and therapeutic supplement. Consequently, this study aimed to comprehensively evaluate the impact of dietary bee venom-loaded chitosan nanoparticles (BV-CSNP) as a novel growth promoter on the health of Pacific white shrimp (*Litopenaeus vannamei*). The investigation encompassed an assessment of growth parameters, whole-body composition, non-specific immune indicators, the transcriptional expression of antioxidants and immune-related genes, organ histology, and disease resistance against a *V. parahaemolyticus* challenge.

## Materials and methods

### Sources of Bee-Venom and chitosan

Fresh exuviae shells from *Hermetia illucens* (AMININS Co., Egypt) were washed with distilled water, then dried in an oven at 70 °C for 36 h. After drying, the shells were crushed using a blender and stored at room temperature for future use. Chitosan production commenced with preconditioning the shells to remove loosely bound proteins. One hundred grams of shells were immersed in a 0.05 M acetic acid solution (Sigma-Aldrich Co., USA) for 24 h, followed by washing with distilled water (D.W) and drying in an oven at 70 °C for 4 h. The next stage, deproteination, involved dissolving proteins and sugars. The preconditioned powder was soaked in a 12.5 M NaOH solution (1 part shell to 15 parts solution by w/v) for 2 h under constant magnetic stirring, then cooled at room temperature for 30 min. Next, demineralization entailed filtering the shells, washing the residues with D.W until neutral pH, and re-drying them. Subsequently, the resulting powder was treated with 1 L of 1% HCl for 24 h. After filtration, the residue was washed with D.W until neutral pH and dried in an oven at 70 °C for 4 h. The final step, deacetylation, partially removed acetyl groups from the chitin structure. This process involved adding a 50% NaOH solution (1 part shell to 10 parts solution w/v) and stirring magnetically at 100 °C for 6 h. The sample was then cooled to room temperature, filtered, washed with D.W until neutral pH, and dried in an oven at 70 °C. The resulting material was chitosan^31,32^.

### Synthesis of Bee-Venom loaded chitosan nanoparticles (BV-CS NPs)

Chitosan nanoparticles (CSNPs) were fabricated using the ionotropic gelation method^33^. First, 0.3 g of chitosan was dissolved in 100 mL of 1% acetic acid (in D.W) and magnetically stirred at room temperature until a clear solution was obtained. The chitosan solution’s pH was then adjusted to 5 with 1 M NaOH. Concurrently, a sodium tripolyphosphate (TPP) solution (Sigma-Aldrich Co., USA) was prepared by dissolving 0.1 g of TPP in 33.3 mL of D.W. This TPP solution was added dropwise to the chitosan solution under magnetic stirring at 1000 rpm at room temperature, maintaining a 3:1 (v/v) chitosan: TPP ratio. Sonication was performed for 20 min. The resulting chitosan particle suspension was cooled, centrifuged at 12,000 rpm for 60 min, lyophilized, and stored at 4 °C. For bee venom-loaded nanoparticles, 0.1 g of honeybee venom (purchased from the Department of Honeybee Research, Institute of Plant Protection, Giza, Egypt) was added to the TPP solution *before* its addition to the chitosan solution. Finally, the mixture was centrifuged at 11,000 rpm and 4 °C for 60 min, lyophilized, and stored at 4 °C. All chemicals and solvents used were of analytical grade and high purity.

### Characterization of BV-CSNPs

The morphology of CSNPs and BV-CSNP (combination) was identified. For TEM analysis, a drop of each sample was placed on a carbon-coated copper grid and allowed to dry at room temperature. Electron micrographs were acquired using a JEOL JEM-1010 transmission electron microscope operating at 80 kV at the Regional Center for Mycology and Biotechnology (RCMB), Al-Azhar University^34^.

### Shrimp and rearing conditions

After two weeks of acclimation, 240 healthy *L. vannamei* with an average initial weight of 6.00 ± 0.02 g were randomly selected and uniformly distributed into four triplicate groups in 1m^3^ hapas (20 shrimp/hapa). This took place at a private earthen pond shrimp farm in Damietta, Egypt. Throughout the experimental period, the shrimp rearing conditions maintained the following water parameters: dissolved oxygen 6.60 ± 0.03 mg/L, pH 8.16 ± 0.01, salinity 37.50 ± 0.04 g/L, ammonia nitrogen content 1.22 ± 0.01 mg/L, toxic ammonia 0.13 ± 0.00, and temperature 27.13 ± 0.07 °C. Shrimp were fed daily at 5% of their body weight, with 15% of the rearing water changed daily.

### Diet Preparation and feeding protocol

- The nutritional ingredients and chemical composition of these experimental diets are detailed in Table 1. We prepared experimental diets by thoroughly mixing a commercial shrimp diet (Skretting Company, Egypt^®^) with various supplements. Chitosan nanoparticles (CS NPs) were incorporated at a concentration of 1 g per kg of diet, based on established protocols^26^. Bee venom loaded chitosan nanoparticles (BV-CSNPs) was then added to the commercial diet at three different concentrations: 0.1, 0.2, and 0.3 mg per kg of diet. A control group received only the basal commercial diet without any supplementation. Shrimp were fed these diets three times daily, at 8:00 AM, 12:00 PM, and 4:00 PM, for a period of 63 days. The daily feeding rate was maintained at 5% of their total body weight.

**Table 1:** The proximate composition and formulation of experimental diets with BV-CS NPs (mg/kg) for Pacific white shrimp, *L. Vannamei.*

Ingredient	Experimental diets (g/kg)			
	Control	Diet 1	Diet 2	Diet 3
Soybean meal	150.0	150.0	150.0	150.0
Shrimp meal	250.0	250.0	250.0	250.0
Rice bran	70.0	70.0	70.0	70.0
Wheat flour	119.0	118.9	118.8	118.7
Fish meal	300.0	300.0	300.0	300.0
Fish oil	60.0	60.0	60.0	60.0
CS NPs	1.0	1.0	1.0	1.0
BV-CSNP (mg/kg)	0.0	0.1	0.2	0.3
CMC^1^	10.0	10.0	10.0	10.0
Vit.mix^2^	20.0	20.0	20.0	20.0
Min mix^3^	20.0	20.0	20.0	20.0
**Total**	1000.0	1000.0	1000.0	1000.0
**Chemical composition**				
Moisture	90.36	90.02	90.77	90.32
Crude protein (Nx6.25)	387.30	387.50	387.70	388.00
Crude fat	109.50	108.20	108.60	108.70
Crude Fiber	17.20	16.70	16.20	16.20
Ash	67.50	66.20	67.10	67.20
NFE	331.20	332.40	332.20	331.80

^1^CMC= Carboxymethyl Cellulose. ^2^Mineral mix (g/kg of premix): MgCO_4_.7H_2_O, CaHPO_4_.2H_2_O, 727.2; 127.5; KCl 50.0; NaCl, 60.0; ZnCO_3_, 5.5; FeC_6_H_5_O_7_.3H_2_O, 25.0; Cu (OAc)_2_.H_2_O, 0.785; MnCl_2_.4H_2_O, 2.5; CoCl_3_.6H_2_O, 0.477; CrCl_3_.6H_2_O, 0.128; CaIO_3_.6H_2_O, 0.295; Na2SeO_3_, AlCl_3_.6H_2_O, 0.54; 0.03.^2^Vitamin mix (per kg of premix): thiamine, 2.5 g; inositol, 2.5 g; riboflavin, 2.0 g; pyridoxine, 100.0 g; pantothenic acid, biotin, 0.3 g; 100.0 g; folic acid, 0.75 g; 0.005 g; a-tocopherol acetate, 2.0 g; choline, 200.0 g; para-aminobenzoic acid, 2.5 g; nicotinic acid, 10.0 g; cyanocobalamine, retinol palmitate, 20.1 g; menadione, 100,000 IU; cholecalciferol, 500,000 IU.

^3^NFE = Nitrogen Free Extract [1000- (Moisture+Protein+Fat+Ash)].

### Growth performance

For assessing growth attributes, shrimp (20 shrimp) in each group were weighted before and after 63 days after the feeding. The feed conversion ratio (FCR), final weight (FW), specific growth rate (SGR), and weight gain (WG) were determined using the following formulas.

- WG (%) = (Final weight (g)-Initial weight (g))/(Initial weight (g)) ×100.
- SGR= (ln FW - ln IW)/(t)×100.

Where: ln is the natural logarithmic of FBW and IBW; t = time in days.

- FCR= (Feed intake (g))/(weight gain (g)).

### Shrimp body composition analysis

Shrimp body samples (*n* = 3) were evaluated for their proximate components by a conventional method^35^. The Kjeldahl method, which traps NH_3_ with boric acid, was used to calculate crude protein (*N* × 6.25). Dry matter was assessed by drying samples in an oven at 105 °C. The ash concentration was established by burning 2 g of samples in a muffle furnace at 550 °C for 4 h. The Soxtect System HT extraction unit extracted the total lipids with ether. Additionally, gross energy was established through a Schimadzu CA-4P bomb calorimeter^36^.

### Hemolymph sampling and non-specific immunological parameters analysis

Hemolymph samples (250 µl) were picked up from five randomly selected shrimp per treatment from the ventral-sinus cavity using a 25-gauge needle and a 1 ml syringe that was inserted with a pre-cooled (4 °C) anticoagulant^37^.

#### Total hemocyte count

Fifty µl of the anticoagulant-hemolymph mixture was diluted in 150 µl of formaldehyde (4%). Then 20 µl of the diluted mixture was examined on a Neubauer hemocytometer to measure the total hemocyte count, THC. Hemocytes were counted using a light microscope at 100× magnification and calculated as; THC (cells/mL) = count×10^6^ × dilution factor.

#### Phenoloxidase activity

Phenoloxidase (PO) activity was determined spectrophotometrically by monitoring the production of dopachrome from L-dihydroxyphenylalanine (L-DOPA)^37,38^. The shrimp’s PO activity was assessed by measuring the absorbance of dopachrome at 490 nm, which was quantified by observing the increase in optical density per minute per milliliter of protein.

#### The lysozyme activity

Lysozyme activity (U ml^−1^) was evaluated in shrimp hemolymph using the ELISA micro-well technique with a lysozyme ELISA kit (CAT. No. SL0050FI, Sunlong Biotech Co., China). Following the manufacturer’s instructions, the reaction was carried out at room temperature, and the absorbance at 450 nm was recorded using a microplate ELISA reader at 30 s and 4 min. One unit of lysozyme activity was defined as the amount of sample needed to cause a decrease in absorbance by 0.01 per minute^39^.

#### The phagocytotic activity

The phagocytotic assay followed the procedures of a previous study^40^ with some modifications. Shrimp hemocytes were cleansed with a saline solution (NaCl 28.4 g, MgCl2-6H2O 1.0 g, MgSO4-7H2O 2.0 g, CaCl2-2H2O 2.25 g, KCl 0.7 g, glucose 1.0 g, and HEPES 2.38 g/l) and the viable cell count was adjusted to 1 × 10^6^ cells/ml. The cell suspension (200 µl) was applied to a cover slip. The cell suspension was withdrawn after 20 min and thoroughly washed with shrimp saline 3 times. The *Candida albicans* preparation (2 mL) was added and incubated for 2 h. Afterwards, the cell suspension was rinsed 5 times with shrimp saline to attain a concentration of 5 × 10^8^ cells/ml, then fixed with 100% methanol. The cover slip was stained with Giemsa stain, mounted and examined under the microscope. Two hundred hemocytes were counted for each sample. The phagocytic activity and index were calculated using the following equation.

-Phagocytic rate (PR) (%) = (Number of phagocytic hemocytes)/(Total number of hemocytes)×100,
-Phagocytic index (PI)= (The Number of phagocytic *C. albicans* cells by hemocytes)/(Total number of phagocytes hemocytes) ×100.

**Bactericidal activity**: Bactericidal activity was determined as described by Adams^41^. Hemolymph samples were serially diluted in a sterile buffer saline (pH 7.5) at 1:2, 1:4, 1:8, 1:16, and 1:32. *V. parahaemolyticus* suspensions were appropriately diluted with their respective saline diluents before 100 µl of each bacterial suspension was incubated with 100 µl of hemolymph, which was incubated at room temperature for 3 h. Fifty microliters of each hemolymph-bacterial mixture were then inoculated onto TSA plates and incubated at 28 °C for 24 h, alongside a positive control plate. Colony-forming units (CFU) were counted using the plate counting method, and the results were expressed as the percentage inhibition (PI) of *V. parahaemolyticus* was calculated as follows:

Inhibition % = [100- (mean CFU in sample/mean CFU positive control)] × 100.

### Antioxidant activity assessment

Superoxide dismutase (SOD) activity was measured using a commercial kit (Biodiagnostic Co., Egypt; SD 2521) according to the manufacturer’s instructions. Absorbance was measured calorimetrically at 560 nm^42^, and SOD activity was expressed as units per milliliter (U/mL). Catalase (CAT) activity was measured at 550 nm using a microplate reader, following the method described by Johansson and Håkan Borg^43^, and expressed as units per milligram of protein (U/mg protein). Malondialdehyde (MDA) levels were measured according to a previously described method^44^, and the lipid peroxide level was expressed in µmol l ^−1^.

### Digestive enzyme activity

Lipase and amylase activities were measured colorimetrically for each treatment. Intestinal samples (*n* = 3) were homogenized in sterile, ice-cold saline solution and centrifuged at 3500 × g for 10 min at 4 °C. Supernatants were collected for analysis. Lipase activity was determined using a commercial kit (Spectrum, Egypt; REF: 281 001) and expressed as units per milligram of protein (U/mg protein)^45^. Amylase activity was measured using a commercial kit (Biodiagnostic Co., Egypt; CAT. NO. AY 10 50) according to the manufacturer’s instructions and expressed as U/mg protein. Protease activity was measured using a fluorometric protease activity assay kit (ab112152).

### Transcriptional expression of antioxidant and immune-related genes

Total RNA was isolated from the hepatopancreas tissues of each experimental group using the RNA purification kit (Thermo Fisher Scientific, USA) according to the manufacturer’s instructions. A nanodrop lite spectrophotometer (Thermo Scientific, US) was used to measure the O.D. 260 nm/O.D. 280 nm ratio to assess the quality of the extracted RNA. Complementary DNAs (cDNAs) were synthesized from 1 µg of RNA using the SuperScript TM III First-Strand Synthesis System (Invitrogen, USA) and Oligo-dT primers following the manufacturer’s instructions. After that, the cDNA samples were stored at −20 °C until they were needed again. Consequently, the mRNA expressions of immune (Lipopolysaccharide and β−1,3-glucan-binding protein (*LGBP*), Peroxinectin (*PX*), Prophenoloxidase activating (*ppA*) enzyme and antioxidant (cytosolic manganese superoxide dismutase (*cytMnSOD*), mitochondrial manganese superoxide dismutase (*mtMnSOD*) related genes were valued using qPCR (SensiFast SYBR Lo-Rox kit, Bioline, London, UK) to quantify the gene transcription folds **(**Table 2**).** The thermal cycle conditions for the reaction were adjusted as follows: 10 min at 95 °C, 40 cycles at 95 °C for 15 s, 30 min at 60 °C, and finally 5 min at 85 °C. The transcription levels were standardized to the β-actin gene as an internal control gene according to the 2^−ΔΔCT^ method^46^.

**Table 2:** The sequence of forward and reverse primers used for q-PCR analysis.

Gene	Primer Sequence 5´ − 3´	Bp	Accession no/ref.
***LGBP***	F: CGG CAACCAGTACGGAGG AAC R: GTGGAAATCATCGGCGAAGGA G	118	XM_027358431.1
***PX***	F: ATCCAGCAGCCAGGTATG R: CAGACTCATCAGATCCATTCC	147	XM_027372426.1
***ppA***	F: CTAGAGACGTCGGTGTCATCA CC R: AACTTGCCGTCCGAAGTGCG	151	AY368151
***cytMn*** **SOD**	F: TGACGAGAGCTTTGGATCATT CC R: TGATTTGCAAGGGATCCTGGTT	155	XM_027376216.1
***mtMn*** **SOD**	F: CAGACTTGCCCTACGATTAC R: AGATGGTGTGATTGATGTGAC	216	XM_027381242.1
***β-actin***	F: CTTGTGTGCGACAATGGCTC R: TCGATGGGGTACTTGAGGGT	194	XM_027371505.1

*LGBP*: Lipopolysaccharide and β-1,3-glucan-binding protein, *PX*: Peroxinectin, *ppA:* Prophenoloxidase, *cyt*Mn*SOD*: cytosolic manganese superoxide dismutase, and *mt*Mn*SOD*: mitochondrial manganese superoxide dismutase.

### Histopathological analysis

The hepatopancreas, intestine, and muscle tissue from shrimp of each treatment group (three shrimp/hapa) were collected after 63 days and fixed in Davidson solution for 24 h. The tissues were then dehydrated in a series of ethanol concentrations (70%, 80%, 90%, and 100%), cleared in xylene, embedded in paraffin, and sectioned at a thickness of 4 mm. The tissue slides were stained with H&E dyes and examined under a light microscope (Leica, UK) for histological evaluation, with images taken for documentation^47^. The villus height (VH), villus width (VW), and intestinal muscle thickness (MT) were measured using the software ImageJ imaging software (1.54d, NIH, USA) and evaluated.

### Challenge assay

Following the feeding trial, a challenge test was conducted to evaluate shrimp resistance to a pathogenic *V. parahaemolyticus* strain^48^. The bacterial strain was cultured in Brain Heart Infusion broth supplemented with 2% NaCl at 28 °C for 24 h. After incubation, the bacterial suspension was centrifuged, and the resulting pellet was washed several times with sterile saline solution. The pellet was then resuspended to a final concentration of 10⁷ CFU/mL. Thirty shrimp from each dietary treatment (Control, T1, T2, and T3) were randomly distributed into twelve tanks (60 × 35 × 30 cm³) with three replicates per treatment (10 shrimp/tank). All groups were challenged by immersion in water containing the *V. parahaemolyticus* suspension at a concentration of 10⁷ CFU/mL for 24 h at 28 °C^49^. Shrimp were monitored for six days post-challenge, or until mortalities ceased. Dead shrimp were removed and recorded daily. *V. parahaemolyticus* was re-isolated from the hepatopancreas of freshly dead shrimp using Thiosulfate-Citrate-Bile Salts-Sucrose Agar (TCBS). Mortality rates were calculated as: Mortality (%) = (Number of dead shrimp/Total number of shrimp) × 100.

### Statistical analysis

To analyze the data and identify significant differences among treatments, we employed one-way ANOVA followed by Duncan’s multiple range test. Statistical analyses were conducted using SPSS version 26.0 (IBM Corp., Armonk, NY, USA). Results are presented as means ± standard error of the mean (SEM), with statistical significance set at *P* < 0.05.

## Results

### Characterization of CSNPs and BV-CSNPs

**Fig. 1:**
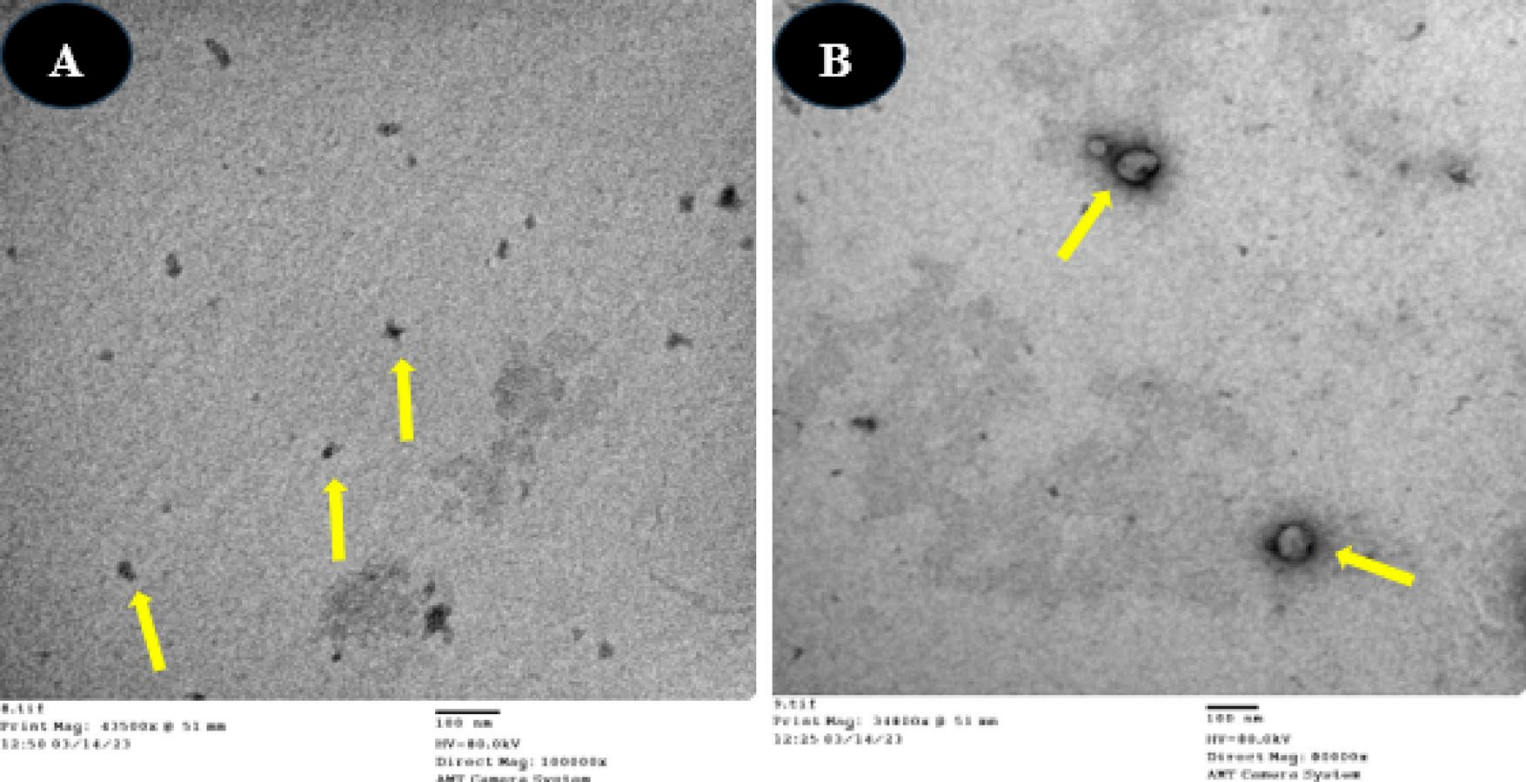
TEM images of the biosynthesized nanoparticles: (**A**) CSNPs, and (**B**) BV-CSNPs. The nanoparticles are clearly visible as distinct, well-dispersed spherical structures. TEM images were captured at a magnification of 48.000×.

TEM screening was employed to examine the particle size distribution and morphology of both chitosan nanoparticles (CSNPs) and bee venom-loaded chitosan nanoparticles (BVCSNPs). TEM images were captured at a magnification of 48.000×. The analysis showed that CS NPs were spherical with smooth surfaces and had an average size of 39.5 ± 8.21 nm. In contrast, BV CS NPs exhibited an average size of 98.25 ± 7.22 nm (Fig. 1).

### Growth and survival

After a 63-day feeding trial, shrimp growth performance was evaluated, and the results are summarized in Table 3. Shrimp fed diets containing varying concentrations of BV-CSNPs exhibited significantly enhanced FW, WG, SGR, and biomass compared to the free diet of BV-CSNPs group (*P < 0.05*). Furthermore, the FCR was notably lower in all BV-CSNP-supplemented groups compared to the control (*P* < 0.05). Interestingly, there were no significant differences in FCR observed among the various BV-CSNP concentrations. It’s also worth noting that survival rates, and FI did not differ substantially among any of the treatment groups (*P < 0.05*). The most favorable growth, as indicated by the highest FW, WG, and average daily gain (ADG), was observed in shrimp fed the diet containing 0.2 mg/kg BV-CSNPs. Shrimp fed the diet containing 0.2 mg/kg of BV-CSNPs showed significantly greater biomass compared to the control group (*P* < 0.05). However, no significant differences in biomass were observed among the various BV-CS NP-treated groups (*P* > 0.05).

**Table 3:** Growth attributes and feed efficiency of shrimp (*L. vannamei*) fed the control, and the various levels of BV-CSNPs supplemented diets over a 63-day feeding trial (means ± SE).

Parameters	BV-CSNPs level (mg/kg)			
	Control	0.1	0.2	0.3
**IW(g)**	6.00 ± 0.12	5.97 ± 0.07	6.03 ± 0.12	6.07 ± 0.03
**FW (g)**	17.43 ± 0.51^b^	19.17 ± 0.92^ab^	20.35 ± 0.98^a^	19.59 ± 0.29^ab^
**WG (g)**	11.43 ± 0.41^b^	13.20 ± 0.85^ab^	14.32 ± 0.86^a^	13.52 ± 0.26^ab^
**WG (%)**	1.69 ± 0.02^b^	1.85 ± 0.06^a^	1.93 ± 0.05^a^	1.86 ± 0.02^a^
**FI (g)**	18.90 ± 0.36	18.80 ± 0.21	19.01 ± 0.38	19.11 ± 0.11
**FCR**	1.66 ± 0.04^a^	1.43 ± 0.07^b^	1.33 ± 0.06^b^	1.41 ± 0.02^b^
**SGR (%)**	1.69 ± 0.02^b^	1.85 ± 0.06^a^	1.93 ± 0.05^a^	1.86 ± 0.02^a^
**ADG (% day** ^**−1**^ **)**	0.18 ± 0.01^b^	0.21 ± 0.01^ab^	0.23 ± 0.01^a^	0.21 ± 0.00^ab^
**Biomass** **(Kg/m** ^**3**^ **)**	325.15 ± 8.93^b^	383.33 ± 18.41^ab^	393.79 ± 22.63^a^	379.07 ± 18.53^ab^
**SR (%)**	93.33 ± 1.67	100.00 ± 0.00	96.67 ± 1.67	96.67 ± 3.33

IW: initial shrimp weight, FW: final shrimp, WG: weight gain, FI: Feed intake, FCR: Feed conversion ratio, SGR: Specific growth rate (SGR; %/shrimp/day), ADG: Average daily gain, and SR: Survival rate. Means **± **SE with different superscripts in the same row are significantly different at *P < 0.05*.

### Whole body composition

Table 4 presents the whole-body composition of shrimp after a 63-day feeding assessment with diets containing BV-CSNPs and a control diet. Overall, no significant alterations (*P > 0.05*) were observed in moisture, dry matter, protein, or lipid content between the BV-CSNPs-fed groups and the control. The shrimp fed 0.1 mg BV-CSNPs exhibited meaningfully higher moisture content and lower dry matter and protein content. In contrast, ash content was drastically greater (*P < 0.05*) in all BV-CSNP-fed groups when compared to the basal diet. Interestingly, gross energy tended to decrease (*P > 0.05*) with raising amounts of BV-CSNPs in the shrimp diets.

**Table 4:** Carcass proximate composition (%) of shrimp, *L. vannamei* fed the control and the various levels of BV-CSNPs supplemented diets over a 63-day feeding trial (means ± SE).

Parameters	BV-CSNPs level (mg/kg)			
	Control	0.1	0.2	0.3
**Moisture (%)**	75.99 ± 0.03^b^	76.71 ± 0.13^a^	76.03 ± 0.06^b^	75.91 ± 0.15^b^
**DM (%)**	24.01 ± 0.03^a^	23.29 ± 0.13^b^	23.97 ± 0.06^a^	24.09 ± 0.15^a^
**Protein (%)**	16.47 ± 0.07^a^	16.13 ± 0.20^ab^	16.00 ± 0.06^b^	16.07 ± 0.09^ab^
**Lipid (%)**	1.64 ± 0.02	1.63 ± 0.01	1.61 ± 0.00	1.60 ± 0.01
**Ash (%)**	3.04 ± 0.03^b^	3.13 ± 0.01^a^	3.15 ± 0.01^a^	3.16 ± 0.02^a^
**Gross Energy (Kcal/kg)**	3.23 ± 0.02^a^	3.08 ± 0.03^b^	2.95 ± 0.03^c^	2.49 ± 0.03^d^

DM: Dry matter. Means ± SE (*n *= 3 shrimp/replicate) with different superscripts in the same row are significantly different at *P < 0.05*.

### Non-Specific immunological parameters

Table 5 exhibits the immunological parameters of shrimp after 63 days of feeding with experimental and control diets. A significant increase (*P* < 0.05) in total hemocyte count was noted in the hemolymph of shrimp fed diets supplemented with BV-CSNPs, with higher dietary inclusion levels leading to greater counts. Furthermore, phenoloxidase activity was significantly elevated (*P < 0.05*) in shrimp fed 0.3 mg of BV-CSNPs compared to all other groups. However, there were no significant differences were observed among the other BV-CSNPs groups. Lysozyme activity was significantly enhanced (*P < 0.05*) in the group fed the highest level of BV-CSNPs (0.3 mg/kg) compared to the control and other BV-CSNPs groups. Shrimp fed the diet containing 0.3 mg/kg of BV-CS NPs exhibited significantly higher phagocytic activity compared to both the control group and all other treatment groups. The phagocytic index was significantly higher (*P < 0.05*) in shrimp fed 0.2 mg/BV-CSNPs linked to the control and other BV-CSNPs groups. Furthermore, hemolymph from all shrimp fed BV-CSNP diets exhibited significantly greater bactericidal activity (*P* < 0.05) compared to the control group. This indicates an improved capacity to combat bacterial infections. Within the BV-CSNP groups, the shrimp in 0.3 group demonstrated the highest bactericidal activity, suggesting a dose-dependent effect on this immune response.

**Table 5:** Immune-related parameters of Pacific white shrimp, *L. vannamei* fed the control and the various levels of BV-CSNP supplemented diets at 63 days feeding trial (means ± SE).

Immunity Indexes	BV-CSNPs level (mg/kg)			
	Control	0.1	0.2	0.3
**THC (×10**^**6**^ **cell/mL)**	1.99 ± 0.05^d^	2.67 ± 0.20^c^	3.52 ± 0.14^b^	4.90 ± 0.14^a^
**PO (U/mg protein)**	5.28 ± 0.17^b^	5.85 ± 0.21^ab^	6.26 ± 0.32^ab^	6.76 ± 0.51^a^
**LYZ (U/mg protein)**	1.43 ± 0.16^b^	2.01 ± 0.08^b^	2.00 ± 0.10^b^	5.78 ± 0.80^a^
**PA (%)**	7.36 ± 0.35^b^	8.54 ± 0.41^b^	8.55 ± 0.31^b^	10.68 ± 0.78^a^
**PI (%)**	1.11 ± 0.13^b^	1.17 ± 0.11^b^	2.10 ± 0.11^a^	1.46 ± 0.16^b^
**BA (% inhibition)**	25.55 ± 0.49^c^	30.97 ± 0.95^b^	32.73 ± 0.40^b^	39.38 ± 1.05^a^

THC: Total hemocyte count, PO: Phenoloxidase activity, LYZ: lysozyme activity, PA: Phagocytic activity, PI: Phagocytic index, and BA: Bactericidal activity. Means ± SE (*n *= 3 shrimp/replicate) with different superscripts in the same row are significantly different at *P < 0.05*.

### Antioxidants status and lipid peroxidation

**Fig. 2:**
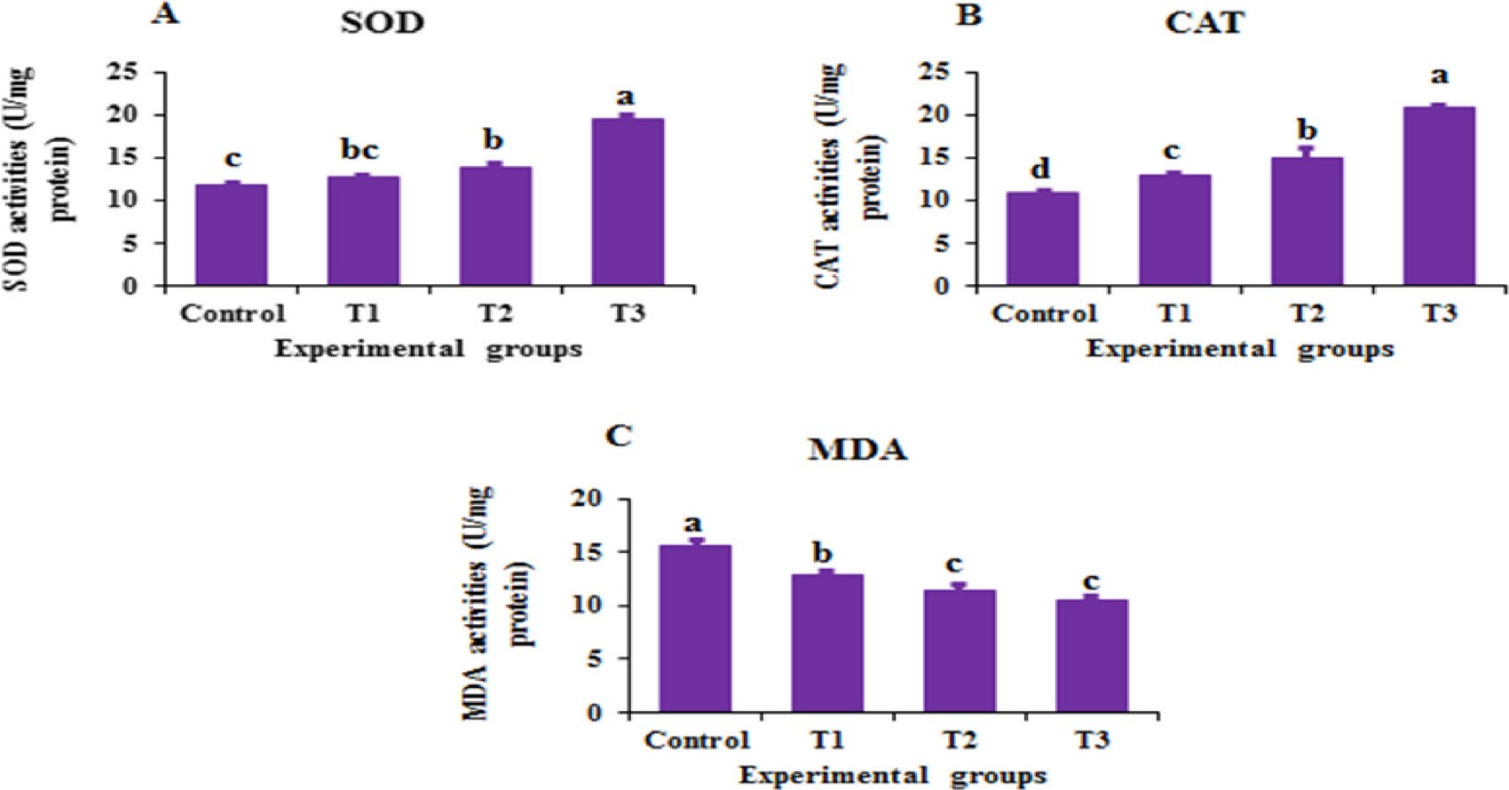
Antioxidant parameters of Pacific white shrimp, *L. vannamei* fed the control and the various levels of BV-CS NPs supplemented diets after 63-day feeding trial (means ± SE). **A**: SOD activity, **B**: CAT activity, and **C**: MDA activity. Superscripts represent significant (*P < 0.05*) differences among treatments.

As shown in Fig. 2, supplementation with 0.2 mg/kg (T2) and 0.3 mg/kg BV-CSNPs (T3) led to a significant increase (*P* < 0.05) in the activities of both superoxide dismutase (SOD) (Fig. 2A) and catalase (CAT) (Fig. 2B) compared to the control and T1 groups. The CAT level was highest in shrimp fed diets containing 0.3 mg/kg of bee BV-CSNPs, significantly surpassing (*P* < 0.05) all other experimental groups. Furthermore, both the 0.2 mg/kg (T2) and 0.3 mg/kg BV-CSNPs groups exhibited a significant reduction (*P* < 0.05) in malondialdehyde (MDA) levels (Fig. 2C) compared to the control.

### Digestive enzymes

Lipase activity (Fig. 4 A) was significantly higher in shrimp fed diets containing 0.2 mg/kg (T2) and 0.3 mg/kg (T3) of BV-CSNPs compared to other groups (*P < 0.05*). The highest levels of lipase activity were observed in the 0.3 mg/kg BV-CSNP (T3) group. The addition of gradual levels of BV-CS NPs to the diet led to a dose-dependent improvement in protease activity (*P < 0.05*), with the highest levels of protease activity were observed in the 0.3 mg/kg BV-CSNP (T3) group (Fig. 4 C). Both the 0.2 mg/kg (T2) and 0.3 mg/kg (T3) concentrations of BV-CSNPs resulted in significantly higher amylase levels (*P* < 0.05) compared to the other experimental groups (Fig. 4B).

**Fig. 3:**
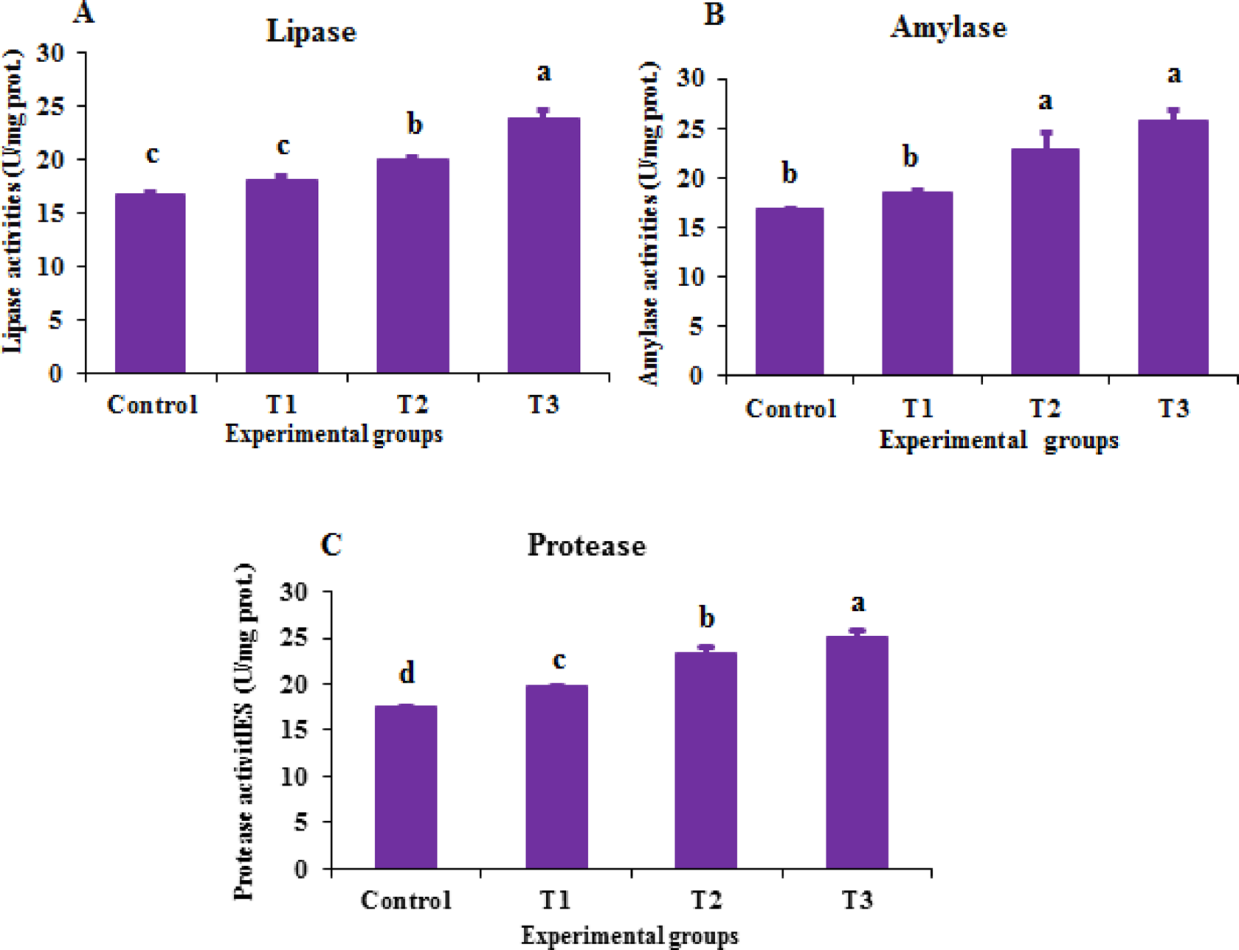
Digestive enzyme activity of Pacific white shrimp, *L. vannamei* fed the control and the various levels of BV-CS NPs supplemented diets after 63-day feeding trial (means ± SE).**A**: lipase activity, **B**: amylase activity, and **C**: Protease activity. Superscripts represent significant (*P < 0.05*) differences among treatments.

### Gene expression

**Fig. 4:**
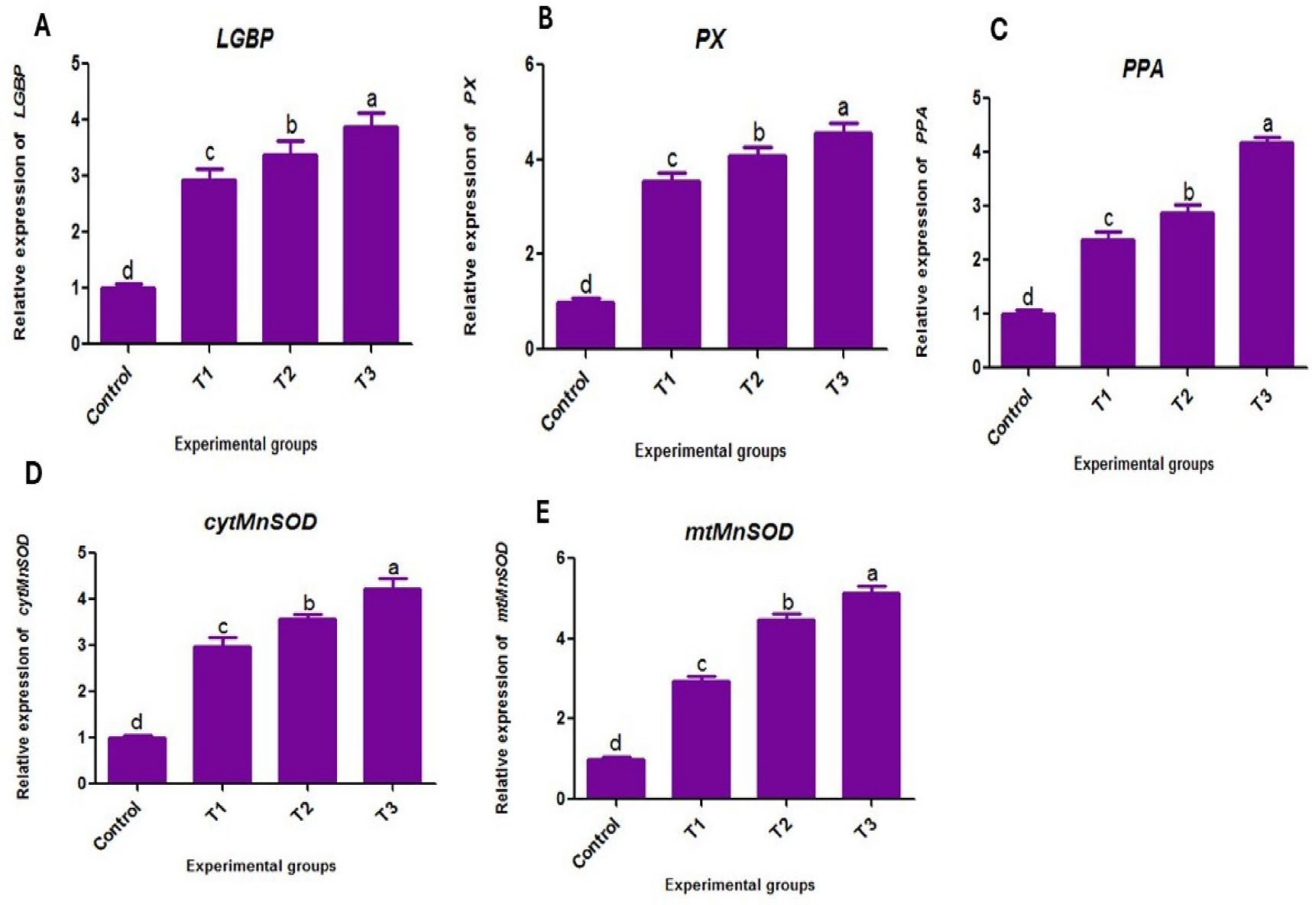
The relative mRNA expressions of *LGBP*, *PX*, *PPT*, *mtMnSOD*, and *cytMnSOD* genes among Pacific white shrimp, *L. vannamei* fed the control and the various levels of BV-CS NPs supplemented diets after 63-day feeding trial (means ± SE). Superscripts represent significant (*P < 0.05*) differences among treatments.

Gene expression analysis (Fig. 4) revealed a significant up-regulation (*P < 0.05*) of *LGBP (*Fig. 4A*)*, *PX (*Fig. 4B*)*, and *PPT* (Fig. 4C) genes in all groups of shrimp fed diets containing BV-CS NPs compared to the control group, showing a dose-dependent response. Similarly, *cytMnSOD* (Fig. 4D) and *mtMnSOD* (Fig. 4E) genes were significantly up-regulated (*P < 0.05*) in all BV-CS NPs-supplemented groups compared to the control group.

### Histopathological analysis

**Fig. 5:**
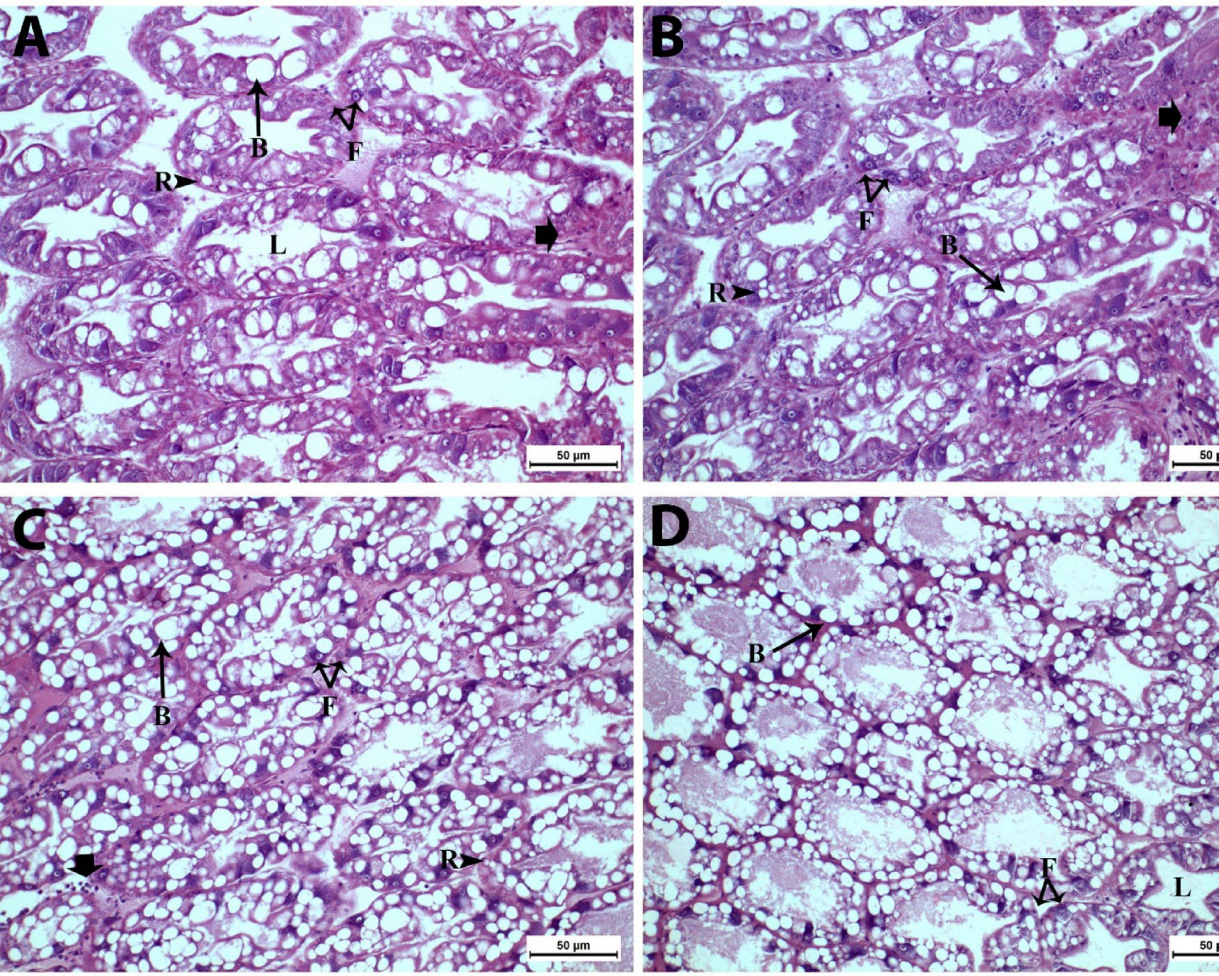
Photomicrograph of hepatopancreas tissues of *L. vannamei* fed with the control and the three BV-CS NPs diets at 63 days. **(A&B)** Control & T1 groups showing normal histological structure with hemocytes infiltration (Short arrow); besides mild edema between hepatopancreatic tubules; **(C)** T2 group showing normal histological structure with hemocytes infiltration (short arrow); **(D)**T3 group showing obvious increase in B-cells (thin arrow), and R-cells (arrowhead). F-cells (double arrows), L: lumen. Stain H&E. Scale Bars = 50 μm.

Histological examination of shrimp hepatopancreas tissue (Fig. 5) provided insights into the cellular structure and overall health of this vital organ across the different dietary groups. In both the control (Fig. 5A) and T1 (Fig. 5B) groups, the hepatopancreas exhibited a normal cellular structure, with clear identification of R-cells (resorptive cells), B-cells (blister-like cells), and F-cells (fibrillar cells). However, these groups also showed mild edema between the tubules and some hemocyte infiltration, suggesting minor stress or inflammatory responses. In contrast, the T2 (Fig. 5C) and T3 (Fig. 5D) groups displayed a normal hepatopancreatic architecture, characterized by well-defined tubules connected by basophilic connective tissue strands. Notably, these groups, especially the T3 group, showed a noticeable increase in the number of R-cells and B-cells.

**Fig. 6:**
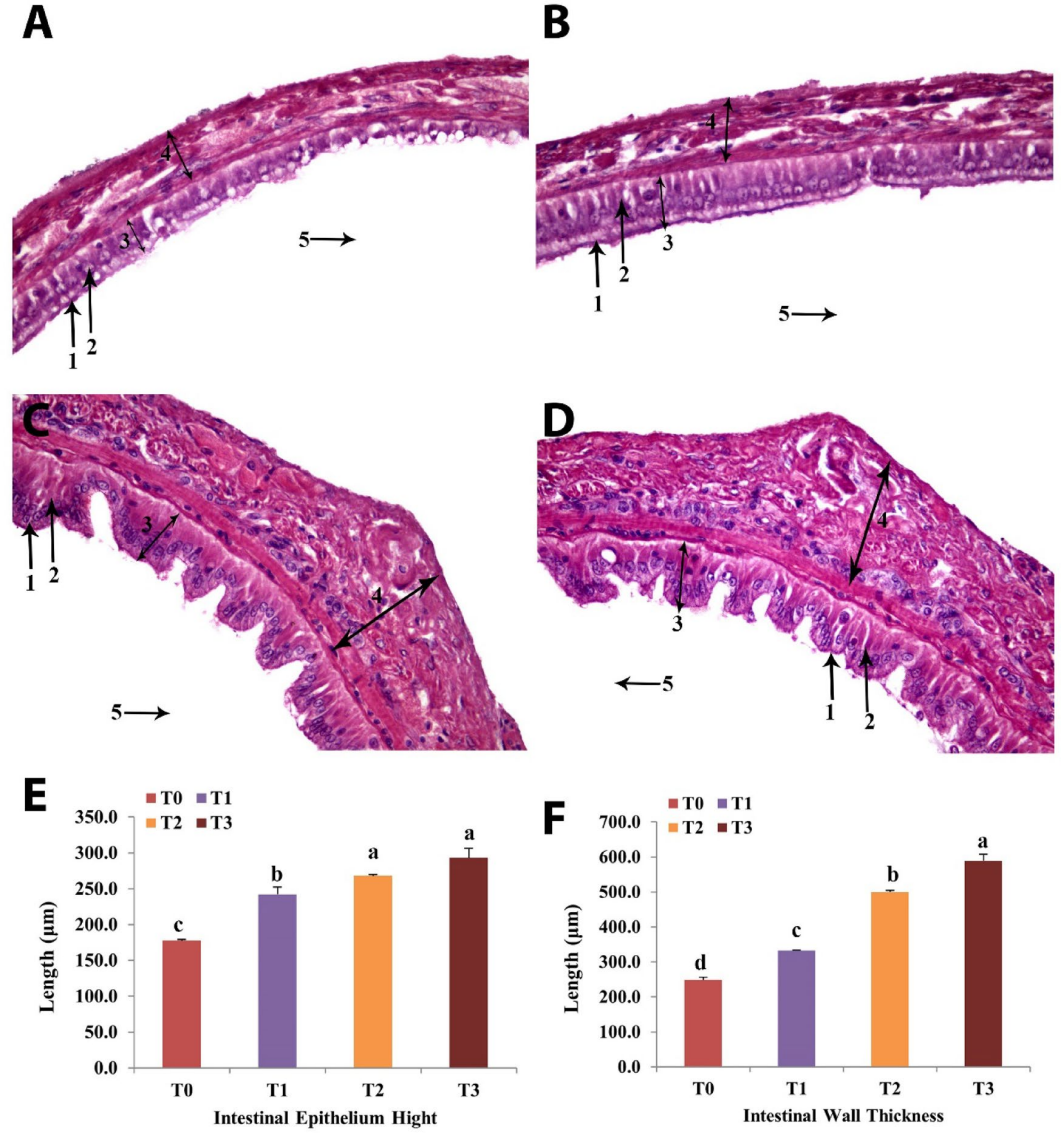
Photomicrograph of intestinal tissues of *L. vannamei* fed the control and the three BV-CS NPs diets at 63 days. **(A)** Control group, T0, showing short epithelium with a thin circular muscle layer besides sloughing of some enterocytes; **(B)** T1 group showing intact epithelium with thin circular muscle layer; **(C& D)** T2 & T3 groups showing increased microvilli folding with improved enterocyte height with thick intestinal wall. **(E)** Enterocytes height, **(F)** Intestinal wall thickness. 1: Mucosa brush border, 2: Intestine epithelium, 3: Epithelium height, 4: Wall thickness, and 5: Intestine lumen. Stain H&E. Scale Bars = 50 μm.

**Fig. 7:**
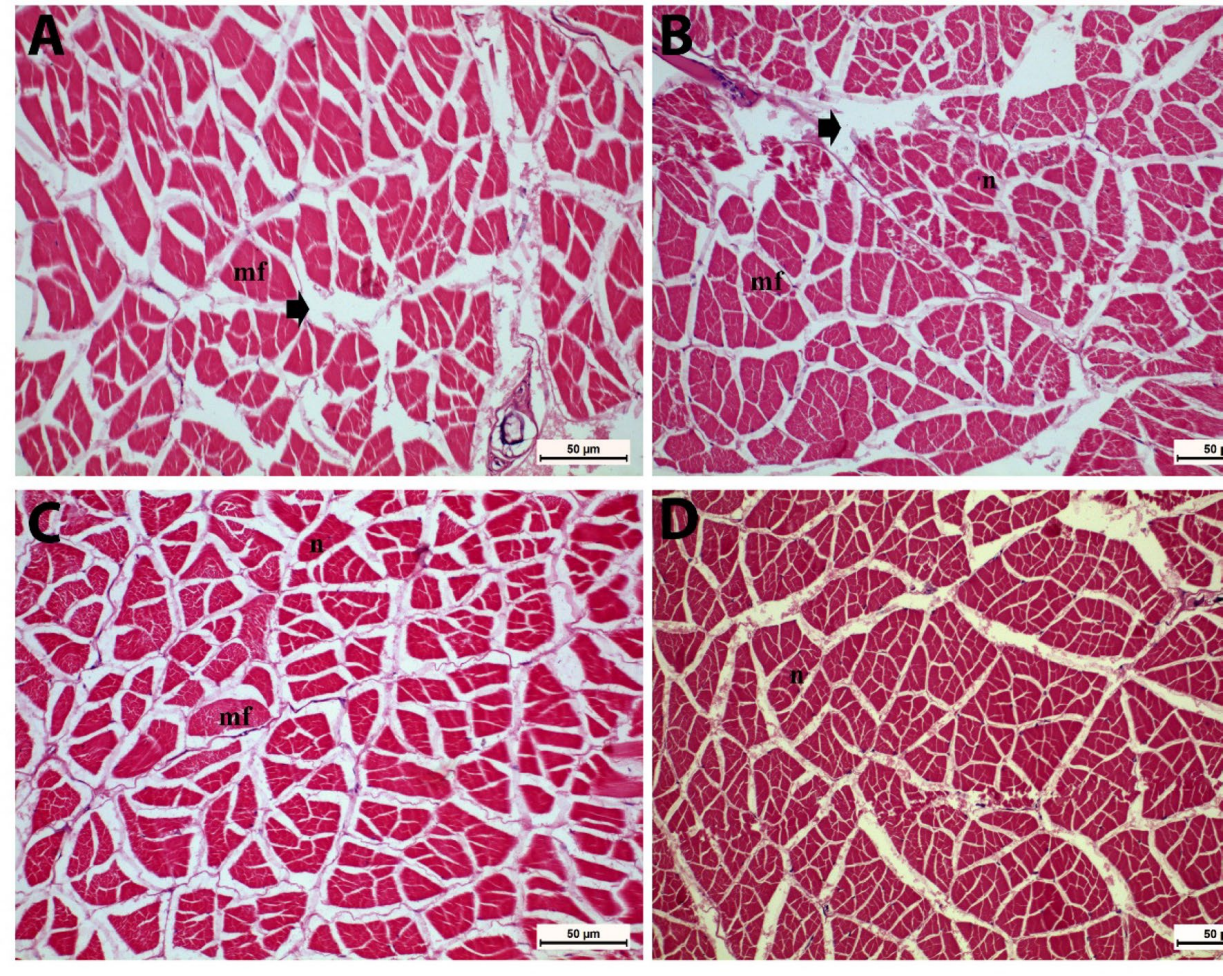
Photomicrograph of muscular tissues of *L. vannamei* fed the control and the three BV-CS NPs diets at 63 days. **(A)** The control and the T1 groups showing intramuscular oedema (black arrow), **(C)** The T2 group showing mild oedema between muscle bundles, and **(D)** The T3 group exhibits normal tissue appearance. mf: muscle fibres, n: muscle nucleus. Stain H&E. Scale Bars = 50 μm.

As shown in Fig. 6 (A-D), shrimp fed BV-CSNP (0.2 or 0.3 mg/kg) diets exhibited an improved intestinal structure compared to the control group (Fig. 6A) and T1 (Fig. 6B). Specifically, the T2 (Fig. 6C) and T3 (Fig. 6D) groups displayed normal epithelium and microvilli morphology. Furthermore, these groups showed significantly greater (*P* < 0.05) enterocyte height (EH) and intestinal wall muscle thickness (WT) when compared to the control group (Fig. 6E & F). These findings suggest enhanced nutrient absorption and overall intestinal health with BV-CS NP supplementation. Regarding muscle tissue, mild intramuscular edema was observed in the control and lower BV-CS NP groups (Fig. 7A-C). In contrast, the T3 group presented normal muscle tissue structure (Fig. 7D), indicating that higher concentrations of BV-CS NPs may help mitigate muscle stress or inflammation.

### Challenge against *V. parahaemolyticus*

**Fig. 8:**
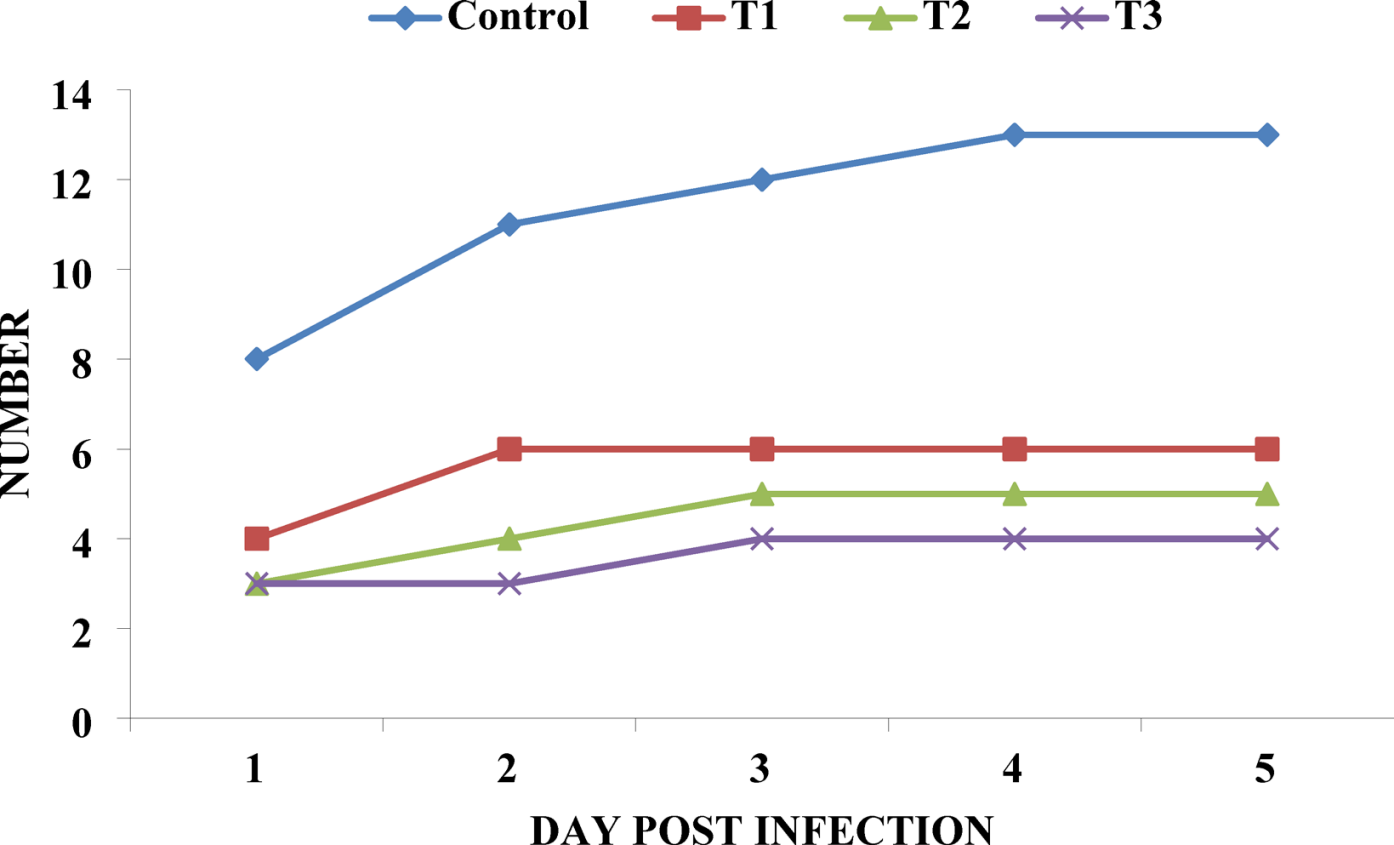
The mortality rate in Pacific white shrimp, *L. vannamei* fed the control and the three BV-CSNPs diets over 63 days and at 5 days post-challenge with *V. parahaemolyticus*.

Following a challenge with virulent *Vibrio parahaemolyticus*, cumulative mortality rates were significantly lower in shrimp fed diets containing BV-CS NPs compared to the control group (Fig. 8). Mortality decreased in a dose-dependent manner, with the lowest mortality rate observed in the T3 group (20%), followed by the T2 group (25%), and then the T1 group (30%). All BV-CS NPs-supplemented groups exhibited substantially lower mortality than the control group (65%).

## Discussion

The pervasive use of antibiotics in aquaculture has led to significant environmental concerns and poses potential risks to both human and aquatic animal health. This pressing issue underscores the urgent need for natural, sustainable alternatives to antibiotics to safeguard the environment while simultaneously boosting productivity and health in farmed fish. Over the past two decades, the exploration of natural molecules has emerged as a promising strategy in aquaculture. However, their efficacy has been dramatically amplified by the integration with cutting-edge advancements in nanotechnology. This synergy allows for enhanced delivery, bioavailability, and targeted action of these natural compounds, paving the way for more environmentally responsible and effective aquaculture practices.

Recent research highlights the significant benefits of various nano-products in aquaculture, offering promising solutions for improving aquatic animal health and productivity. For example, Eissa, et al.^26^ investigated the use of pumpkin seed oil-loaded chitosan nanoparticles in whiteleg shrimp (*Litopenaeus vannamei*). Their findings showed that this nano-product significantly enhanced growth, boosted digestive enzyme activity, improved immune response, and increased survival rates against *V. parahaemolyticus*. Another recent study investigated the effect of BV-CSNPs as a potential therapeutic strategy for enhancing tilapia health and resistance to *Aeromonas hydrophila*^50^. Recent studies have shown that using chitosan-loaded particles in aquaculture can have numerous positive impacts. Likewise, Mabrouk, et al.^51^ found that supplementing the diet of Nile tilapia broodstock with *Arthrospira platensis* nanoparticles led to improvements in growth performance, a more balanced steroid hormone profile, and enhanced reproductive productivity. Similarly, Said, et al.^52^ concluded that dietary selenium nanoparticles derived from *Spirulina platensis* positively impacted growth performance and physio-biochemical components in Pacific white shrimp (*L. vannamei*), while also offering an alleviating effect against cadmium toxicity. Furthermore Eissa, et al.^53^ demonstrated that the dietary administration of nano curcumin to red tilapia (*Oreochromis sp.*) resulted in increased growth performance, improved body composition, healthier blood parameters, beneficial histopathological changes, and enhanced resistance against *Aspergillus flavus*. This experiment is the first to investigate the favorable consequences BV-CSNPs on growth performance, immune response, hemolymph health, organ histology, and survival following challenge with *V. parahaemolyticus*. Dietary supplementation with BV-CSNPs at levels of 0.2 or 0.3 mg/kg improved growth variables and overall health. This enhancement was demonstrated by enhanced antioxidant status (increased SOD and CAT activity), reduced MDA levels, and up-regulated expression of *LGBP*, *PX*, and *PPT* genes.

These improvements can be attributed to the unique characteristics of bee venom, which exhibits promising biological activities. Numerous studies have demonstrated that BV has a unique composition containing a variety of bioactive compounds^54–56^. These compounds contribute to BV’s diverse therapeutic properties, including anti-inflammatory^57^immunostimulatory activity^58^anticancer properties^59^and antimicrobial effects^60^as well as the potential to enhance reproductive^61^ and growth performances^62^.

In the present study, dietary supplementation with BV-CSNPs for 63 days enhanced growth performance in *L. vannamei*, as evidenced by increased final weight and a reduced FCR. Similar improvements in weight gain and survivability have been reported in pigs and broiler chicks supplemented with BV^63^,

This growth enhancement may be attributed to the bioactive compounds in BV, which could improve nutrient absorption and utilization by promoting healthy digestive tract function. This, in turn, would lead to a lower FCR and more efficient growth^64,65^. The observed normal morphology of the intestinal epithelium and microvilli in shrimp fed diets containing higher levels of BV-CSNPs supports this hypothesis. Furthermore, the significant increase in epithelial height and intestinal wall muscle thickness indicates improved intestinal health, which is crucial for nutrient absorption in shrimp^66^. Therefore, dietary BV-CSNPs likely enhance digestive capacity and promote nutrient absorption. These results align with previous research^67^ highlighting the importance of the intestinal mucosa as a primary barrier against pathogens and suggesting that intestinal health significantly influences overall health and immunity in shrimp.

Dietary BV-CSNPs supplementation significantly increased moisture and ash content while decreasing whole-body protein, lipid, and gross energy levels. In a recent study we conducted using red tilapia, we observed similar trends: moisture, protein, and ash content increased, while lipid content decreased. These variations may be attributed to species-specific differences and environmental conditions^68^. Further supporting the positive impact of bee venom, a study in broiler chickens demonstrated that bee venom in drinking water led to increased carcass weight and reduced abdominal fat^69^. Similarly, Kim, et al.^70^ reported that BV supplementation enhanced relative breast meat yield in broiler chickens, although it did not significantly affect abdominal fat weight. These variations could be attributed to the source of bee venom (BV), the study duration, animal type and breed, and other experimental conditions.

This study highlights the immunostimulatory activity of BV in shrimp, demonstrating its positive impact on both cellular and humoral components of the innate immune system. Shrimp fed diets containing BV-CS NPs (0.2 or 0.3 mg/kg diet) showed a significant increase in hemocyte numbers, a key indicator of cellular immune response. This increase was directly correlated with the inclusion level of BV-CSNPs in their diet.

Phagocytic activity of hemocytes was also enhanced in a dose-dependent manner. This rise in phagocytic cells, which are crucial for engulfing and destroying pathogens, aligns with previous research on BV’s effects, such as those observed in male rabbits^61^. The improved immune response likely contributes to greater disease resistance in shrimp^55^. The anti-inflammatory activities of BV are attributed to its bioactive peptides, such as melittin and MCD-peptide, as well as key enzymes, particularly phospholipase A2 (PLA2)^71^. Notably, PLA2 may also play a role in the observed increase in immune cell populations, further bolstering the shrimp’s defense mechanisms^72^. The secondary arm of the shrimp’s innate defense is the humoral response, which involves the release of various immune molecules. This includes the prophenoloxidase (Pro-PO) system^73^and antimicrobial peptides such as penaeidins, and lysozymes, which serve as a first line of defense against invaders, in addition to antioxidant defense enzymes like SOD and CAT play a crucial role in this response^74^.

This study reveals that adding BV to shrimp diets, specifically in the form of BV-CSNPs, can significantly improve their immune system. A key finding is the potential enhancement of phenoloxidase (PO) levels in all shrimp fed BV-CS NPs. PO is a critical enzyme in the proPO-activating system, essential for converting inactive proPO into active PO^75^. This process is vital for melanin biosynthesis, a crucial part of the innate immune response that helps shrimp recognize foreign invaders and activate cellular defenses like hemocyte recruitment, phagocytosis, and melanization. Therefore, higher PO levels directly indicate a stronger immune response. It’s worth noting that a reduction in the proPO system’s activity can impair phagocytosis, underscoring PO’s importance^73^. In addition to PO, the study observed a significant increase in lysozyme activity in shrimp fed the highest concentration of BV-CSNPs. Lysozyme is known to modulate both cellular and humoral defense mechanisms, protecting against diseases. A study by Mai and Wang^76^ confirmed that lysozyme modulated the cellular and humoral defense mechanisms to protect shrimp against diseases. The enhanced immune response in shrimp is likely due to BV’s potent anti-inflammatory and mast cell-degranulating peptides. These peptides stimulate the secretory mechanisms of inflammatory cells, thereby strengthening the overall immune defense^77^.

In this context, BV proves to be a powerful ally in enhancing shrimp health. Its bioactive components, like melittin and adolapin, do more than just scavenge free radicals and reduce oxidative stress. This study shows that in shrimp given high levels of BV-CSNPs, these components significantly ramp up the activity of key antioxidant enzymes such as SOD and CAT. At the same time, they bring down MDA levels, which are indicators of oxidative damage. Essentially, these components work together to neutralize harmful free radicals, lessen oxidative stress, and bolster immune function^56,58^. This protects shrimp cells from damage and keeps them healthy, aligning with findings from various animal studies^61,62^.

This study emphasizes the antioxidant efficacy of BV, evidenced by significantly reduced levels of MDA in shrimp^78,79^. MDA is a widely recognized biomarker for oxidative stress in biological systems^80^. The decrease in MDA indicates that BV effectively protects polyunsaturated fatty acids in cell membranes from lipid peroxidation and subsequent oxidative damage. Therefore, monitoring MDA levels proved to be a crucial indicator for evaluating the effectiveness of dietary antioxidants in mitigating oxidative stress and improving shrimp health.

These findings are further supported by the preservation of normal histological architecture observed in the muscle tissue of shrimp fed high levels of BV-CSNPs. In stark contrast, the control group and those receiving lower doses of BV-CSNPs exhibited clear signs of cellular and tissue degeneration. This provides strong visual evidence of BV’s protective effects against oxidative damage. These findings are corroborated by the preservation of normal histological architecture in the muscle tissue of shrimp fed high levels of BV-CS NPs. In contrast, the control group and those receiving lower doses displayed cellular and tissue degeneration.

This study revealed that shrimp fed diets supplemented with 0.2 and 0.3 mg/kg of BV-CSNPs exhibited significantly higher activity of digestive enzymes, including lipase, amylase, and protease, compared to other groups. These findings support the hypothesis that BV’s immunostimulatory effects contribute to overall shrimp health, leading to improved feed intake and, consequently, enhanced digestive enzyme secretion^81^. Beyond its immune benefits, the anti-inflammatory properties of BV play a critical role in maintaining the integrity of the digestive tract epithelium. This preservation of gut health fosters optimal digestive function, enabling more efficient nutrient digestion and absorption, which ultimately boosts growth performance and overall shrimp health. Further supporting these results is the observation of normal histopathological structure in the hepatopancreatic tissues of shrimp fed BV-CSNPs diets. There was also a notable increase in the number and activity of B-cells, responsible for secreting digestive enzymes, and R-cells, which absorb and store lipids and glycogen.

This study found that shrimp fed diets containing BV-CSNPs showed a significant increase in the expression of several important immune-related genes: LGBP (lipopolysaccharide and β−1,3-glucan binding protein), PX (peroxinectin), and PPA (prophenoloxidase)^82^. LGBP, *PX*^83^and *ppA*^74^ are crucial immune molecules connected to the prophenoloxidase (proPO) system, a vital part of the shrimp’s innate immunity. The upregulation of the LGBP gene in the hepatopancreas of *L. vannamei* shrimp, as observed in this study, aligns with previous research highlighting LGBP’s critical role in shrimp defense. These findings are comparable with prior studies^82,84^ that found the upregulation of the *LGBP* gene in the hepatopancreas of *L. vannamei* and proposed that the *LGBP* has a crucial role in shrimp defense.

Similarly, the *PX* gene of shrimp-fed diets containing BV-CS NPs significantly increased, suggesting that an increase in *PX* can increase the biological activities of cell adhesion^85^opsonin^86^degranulation^87^peroxidase^88^and encapsulation^89^ of shrimp. Moreover, the transcription level of *mt*MnSOD in hepatopancreas was enhanced, indicating that the immune response depending on the ROS system played an important role in shrimp responses against fungal, bacterial, and viral infection, similar to other studies in shrimp^90,91^.

This study found that shrimp fed BV-CSNPs exhibited significantly lower mortality rates after exposure to *Vibrio parahaemolyticus*. This enhanced protection is likely due to dietary supplements improving the shrimp’s gut health and microbial community^92,93^which in turn positively influences the water microbiota.

The inclusion of BV-CS NPs in the diet not only improves survival during a *V. parahaemolyticus* challenge by altering the gut bacterial population but also enhances key immune molecules such as pattern recognition proteins, antimicrobial peptides, and prophenoloxidase. These immune enhancements collectively aid in bacterial clearance and improve infection tolerance, providing a robust defense against pathogens. Adding BV-CSNPs to the diet does more than just help shrimp survive *Vibrio parahaemolyticus* infections. It also improves their ability to fight off bacteria by changing the gut’s bacterial makeup. On top of that, it boosts important immune molecules like pattern recognition proteins, antimicrobial peptides, and prophenoloxidase, all of which help clear out bacteria and increase the shrimp’s tolerance to infections^83^. Further studies are needed to evaluate the economic feasibility of bee venom application in commercial aquaculture and to assess its potential impact on shrimp meat quality.

## Conclusion

This study clearly shows that adding BV-CSNPs to shrimp diets at concentrations of 0.2 or 0.3 mg/kg significantly enhances several key aspects of their health. We observed notable improvements in growth performance, hemocyte count, and the activity of crucial immune enzymes like phenoloxidase and lysozyme. Digestive enzyme activity also increased, alongside positive changes in intestinal histomorphology, indicating better nutrient utilization and gut health. Furthermore, BV-CSNPs led to the upregulation of mRNA expression for important immune genes, including *LGBP*,* PX*,* ppA*,* cytMnSOD*, and *mtMnSOD*. These genetic changes suggest a stronger, more robust immune response. The findings indicate that BV-CSNPs may help mitigate immune-mediated oxidative stress, protecting shrimp cells from damage. Higher levels of BV-CSNP inclusion in the diet appear to strengthen overall immunity, potentially making shrimp more resilient to environmental stressors and pathogen infections. While the use of bee venom in aquaculture is a relatively new area, continued research is highly promising. BV-CSNPs could become a valuable nutritional and therapeutic supplement, especially as a nano-nutrition approach, to improve shrimp health and sustainability in aquaculture.

## Data Availability

All data to support the conclusions have been provided in the manuscript.

## Abbreviations

BV-CS NPs: Bee venom loaded with nano-chitosan
SOD: Superoxide dismutase
CAT: Catalase
HP: Hepatopancreases
SPF: Pathogen-free
IW: Initial shrimp weight
FW: Final shrimp
WG: Weight gain
FI: Feed intake
FCR: Feed conversion ratio
SGR: Specific growth rate
ADG: Average daily gain
SR: Survival rate
THC: Total hemocyte count
PO: Phenoloxidase activity
LYZ: Lysozyme activity
PA: Phagocytic activity
PI: Phagocytic index
BA: Bactericidal activity
ANOVA: Analysis of variance
